# Benign fibrous histiocytoma of parietal bone: case report and review of the literature

**DOI:** 10.1186/s12957-015-0587-5

**Published:** 2015-05-08

**Authors:** Lili Yang, Yan Feng, Xu Yan, Yanhui Li, Li Bie

**Affiliations:** Department of Neurosurgery, First Clinical Hospital, Jilin University, Changchun, China; Department of Obstetrics and Gynecology, First Clinical Hospital, Jilin University, Changchun, China; Department of Radiology, First Clinical Hospital, Jilin University, Changchun, Jilin China; Department of Pathology, First Clinical Hospital, Jilin University, Changchun, Jilin China; Department of Anesthesia First Clinical Hospital, Jilin University, Changchun, Jilin China; Department of Pathology and Laboratory Medicine, University of California, Irvine, USA

**Keywords:** Benign, Fibrous, Histiocytoma, Parietal bone

## Abstract

A benign fibrous histiocytoma with primary site of origin in the parietal bone has not yet been reported in the literature. We report here a case concerning a 12-year-old girl with a 14-month history of an enlarging parietal bone mass. The tumor was excised after removal of the cortical bone and resection of the tumor surrounding the cortical bone erosion using pre-plasticity titanium repair. Both postoperative histopathological examination and immunohistochemical analysis were consistent with a benign fibrous histiocytoma. No clinical or computed tomography (CT) radiological signs of tumor recurrence and/or metastasis were observed at 12 months. Although a primary benign fibrous histiocytoma of the parietal bone is a rare tumor, it should be considered as a potential diagnosis for any cranial tumor. Surgical intervention is the most effective treatment technique for a benign fibrous histiocytoma.

## Background

A benign fibrous histiocytoma (BFH) is a rare tumor of the skeleton and accounts for approximately 1% of all surgically treated benign bone tumors. BFHs of the cranial bones are exceedingly rare and have been described in the skull base, frontal bone, and facial cranium. We report here the first known case of a BFH in the parietal bone, including its diagnosis, surgical excision, and a review of the literature.

## Case presentation

### History

**Figure 1:**
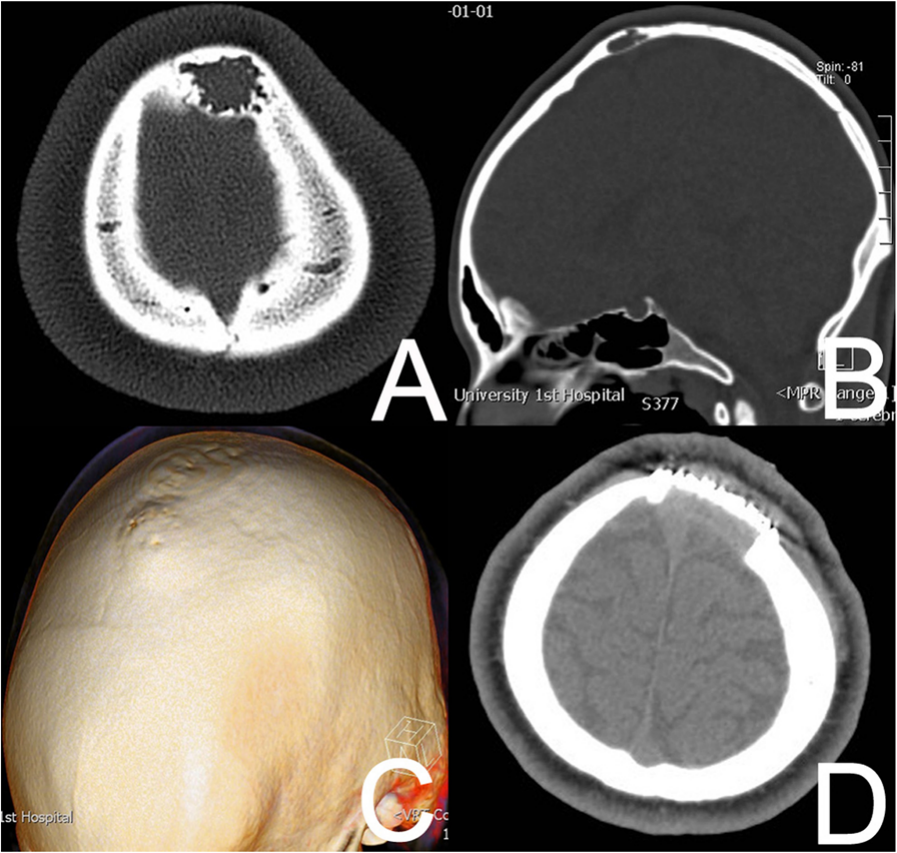
Preoperative CT images of bone-window revealed a low density mass, irregular shape, internal and external bony plates eroded. **(A)** On axial CT scan. **(B)** On sagittal CT scan. **(C)** A 3D reconstructed image by CT showed external bony plates to be jacked up. **(D)** Postoperative CT scan of the axial showing no tumor recurrence 12 months after operation.

A 12-year-old girl presented with a 14-month history of an enlarging parietal bone mass, without pain and cranial nerve deficits. The mass measured 4.0 × 3.0 cm. A computed tomography (CT) scan demonstrated a lesion within the parietal bone mass. The tumor exhibited low density, an irregular shape, and was associated with erosion of internal and external bony plates resulting from osteolytic destruction (Figure 1A, B).

### Operation

Surgery was performed on the patient under general anesthesia, and the external plate of the parietal bone was found to be perforated. The tumor was excised, followed by removal of the cortical bone and resection of the tumor surrounding the cortical bone erosion. Further resection was performed to remove damaged bone from the remaining normal structure of cortical bone, with completion of repair using pre-plasticity titanium mesh. A portion of the tumor, located just above the sagittal sinus, was carefully removed to avoid damage to the sinus during surgery.

### Follow-up

**Figure 2:**
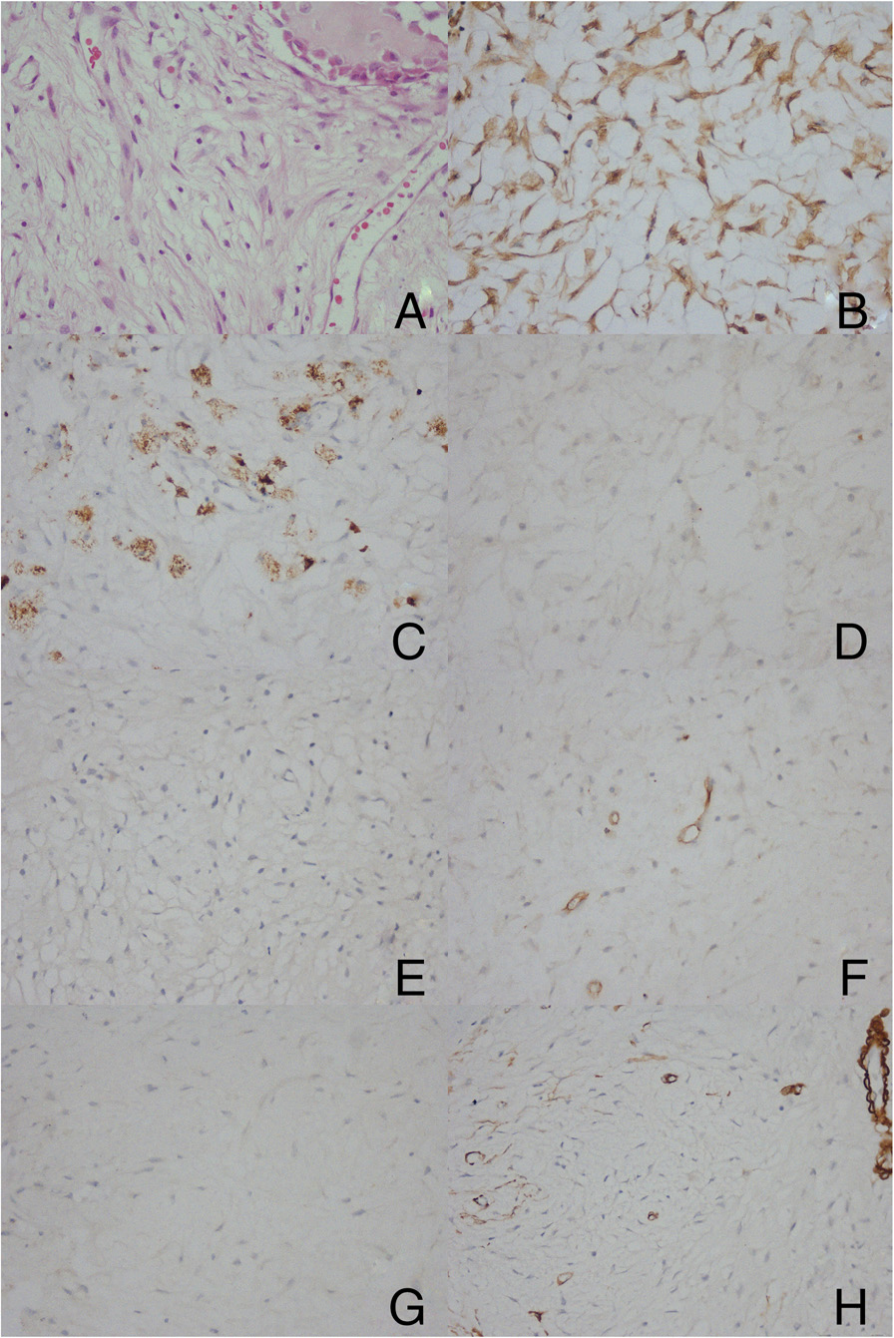
Microphotographs of specimen histology showing the proliferation of spindle-shaped fibroblasts arranged in a storiform pattern. There were some histiocytic foamy cells scattered under the background of the above (hematoxylin and eosin, original magnification ×200) **(A)**. Microphotographs of specimen histology showing the tumor cells positive for Vimentin **(B)**, partially positive for CD68 **(C)**, negative for S-100 **(D)**, GFAP **(E)**, CD34 **(F)**, EMA **(G)** and SMA **(H)**. They confirmed the diagnosis of benign fibrous histiocytoma (immunohistochemical staining, original magnification ×200).

The patient recovered well from the operation. No clinical or CT radiological signs of tumor recurrence and/or metastasis were detected at 6 months and 12 months post-surgery (Figure 1D). Postoperative histopathological examination revealed the proliferation of spindle-shaped fibroblasts arranged in a storiform pattern (Figure 2A). There were some histiocytic foamy cells scattered within the background of the fibroblasts described above. No malignant characteristics, such as nuclear atypia, pleomorphism, or atypical mitosis were observed. Immunohistochemical analysis was performed on the formalin-fixed, paraffin-embedded sections using the streptavidin-biotin-peroxidase complex labeling method. Almost all tumor cells were positive for vimentin, partially positive for CD68, and negative for S100 protein, glial fibrillary acidic protein (GFAP), CD34, epithelial membrane antigen (EMA), and smooth muscle actin (SMA) (Figure 2B,C,D,E,F,G,H). These findings strongly indicate a histiocytic origin for this tumor. Fewer than 1% of cells stained positive for Ki-67 (data not shown).

### Discussion

BFH usually occurs in the dermis, superficial subcutaneous tissue, and deep soft tissues [1]. BFH located in the skeleton is rare, with only about 100 total reported cases. BFHs of bones frequently arise in the flat bones, including the tibia, pelvic bone, and femur [2]. BFH in the cerebral cranium has not been reported, while only four cases have been recorded in the skull base or frontal bone (Table 1). These lesions are more common in males, with a male-to-female ratio of 2.5:1 and tend to occur in young patients with mean age of 37 years [3]. In one report, a BFH was noted in an 11 month-old-girl during drainage of a middle ear infection [4]. The patient in the case report described here was 12 years old.

**Table 1:** **Summary of reported cases of cerebral cranium**

**Number**	**Age (years)**	**Sex**	**Location**	**Interval (months)** ^a^	**Treatment**	**Reference**
1	45	Male	Skull base	2	Surgery	Fritz *et al*., 2006 [6]
2	0.9	Female	Skull base	0^b^	Biopsy	Shane tubbs *et al*., 2007 [4]
3	33	Male	Skull base	2	Surgery	Iedguchi *et al*., 2009[8]
4	22	Male	Frontal bone	15	Surgery	Wen *et al*., 2010 [5]

^a^Number of years between initial be noted of tumor and surgery of the tumor; ^b^tumor was noted during drainage of a middle ear infection.

Patients with BFH of the cranial bone usually present with symptoms which vary with the specific cranial bone location impacted by the tumor. In one report, occlusion of the right transverse sinus in patients with a right occipital protrusion presented with tenderness and horizontal diplopia [2]. Wen *et al.* found that such patients may have symptoms of tumor oppression of the eye [5]. In other cases, if the tumor invaded the pterygopalatine fossa, this has been observed to cause trigeminal nerve hypesthesia, serous otitis media, and decreased mandibular excursion (on the right side in that case) [6]. In the parietal bone BFH tumor described in this report, the tumor exhibited slow growth and was asymptomatic, creating no obvious clinical effects but was found during a shower.

Imaging plays an important role in the diagnosis and identification of BFH. It is our suggestion that CT should be used as a first-line imaging tool in any patient presenting with clinical symptoms of skull tumors. A CT scan usually detects low density or isodensity lesions in bone. BFH shows extra compartmental extensions with somewhat irregular edges and bone defects, as exhibited by thinning of the cortical bone [7]. In contrast, CT scans of typical areas of ground-glass density may confirm the diagnosis of craniofacial fibrous dysplasia [8]. Such lesions are eccentric and multi- or uniloculated and are well demarcated with a sclerotic rim. A diagnosis of nonossifying fibroma can be made with CT scan with an accuracy of 100% [9]. CT images of BFH show a slow-growing tumor such as an expanded intradiploic space, expansive remodeling, and foci of pressure erosion, in some cases, demonstrating osteolytic changes [10]. In our case, the CT scan showed thinning of the cortex, in addition to bone defects. Diagnosis using MRI and PET scans is still in the exploratory stage for detection of BFH. In one pilot study, the tumor in question exhibited homogeneous enhancement by CT with contrast, Gd-enhanced MR imaging, and moderate uptake of FDG in the FDG-PET scan. These imaging methodologies are likely to be very useful for future diagnosis [2].

The lesions in BFH are described as lytic; most exhibit sharply defined margins and a sclerotic rim. The histopathological characteristics of BFH in bone include the proliferation of fibrohistiocytic cells with a storiform or swirl pattern, lipid-filled cells, foam cells, and giant cells that fill the gaps in the fiber network system. Little nuclear atypia, pleomorphism, or necrosis are observed [11]. In our case, fibrohistiocytic cells with a storiform pattern and no nuclear atypia, pleomorphism, or atypical mitosis were observed with a Ki-67 labeling index of <1%.

Complete excision of the tumor is the best treatment option. If lesions are found in the significant area of the skull base, a biopsy could be performed to confirm the diagnosis. However, in the case presented here, the tumor was located in the parietal bone; pathological diagnosis did not affect the tumor resection outcome. Radiotherapy has only been attempted in one case but was only of modest benefit and therefore would play only a very limited role in the treatment of BFH [6].

Follow-up is very important to monitor for tumor recurrence or metastasis. Bielamowica *et al.* reported an 11% recurrence rate after local resection of BFH of the head and neck [3]. Therefore, a CT scan is recommended for follow-up assessment.

## Conclusions

In conclusion, primary BFH of the parietal bone is rarely observed but should be considered as a possible diagnosis of any cranial tumor. Surgical intervention should aim to achieve gross total resection in order to gain the highest chance for a definitive cure. Moreover, radiotherapy and chemotherapy should be individualized to meet the needs of each BFH patient.

### Ethics

The research has been performed in accordance with the Declaration of Helsinki and has been approved by the First Hospital of Jilin University ethics committee.

## Consent

Written informed consent was obtained from the patient for publication of this case report and accompanying images.

## Abbreviations

BFH: benign fibrous histiocytoma
EMA: epithelial membrane antigen
GFAP: glial fibrillary acidic protein
SMA: smooth muscle actin
